# Supplementary material for: VAP‐SCRN1 interaction regulates dynamic endoplasmic reticulum remodeling and presynaptic function
Source: EMBO J. 2019 Aug 23;38(20):e101345. doi: 10.15252/embj.2018101345 (PMC6792018; doi:10.15252/embj.2018101345)
Supplement: Supplementary file 1 — Expanded View Figures PDF [file EMBJ-38-e101345-s001.pdf]

Expanded View Figures

Figure EV1. VAPs modulate bouton maintenance and are associated with brain-enriched SCRN1 proteins.

A Localization of exogenous HA-VAPA or HA-VAPB in neurons (DIV18) co-expressing GFP-Sec61β and immunostained for bassoon. Zooms represent (1) an axonal structure with bassoon-positive presynaptic boutons (arrowheads), and (2) a dendritic structure. Scale bars: 10 μm (full size) and 5 μm (zoom).

B Quantifications of bouton density in hippocampal neurons (DIV18) co-expressing RFP and pSuper empty vector or VAPA/B shRNAs. $N = 2$, $n = 80$ boutons.

C Quantifications of bouton size in hippocampal neurons (DIV18) co-expressing RFP and pSuper empty vector or VAPA/B shRNAs. $N = 2$, $n = 380$–400 boutons.

D Western blot of endogenous SCRN1 expression in indicated adult rat neuronal and non-neuronal tissues. Cereb., cerebellum. Hippoc., hippocampus. Spin., spinal.

E Scaled representation of SCRN1-associated proteins identified with pull-down assay followed by mass spectrometry analysis of purified BioGFP or BioGFP-SCRN1 from HEK293T cell lysates. Selected candidates all showed > 10 enrichment of PSM compared to control.

F Localization of exogenous GFP-SCRN1 in hippocampal neurons (DIV18) immunostained for vGlut. Zoom represents an axon structure with presynaptic sites (arrowheads). Scale bars: 10 μm (full size) and 5 μm (zoom).

G COS7 cells expressing BioGFP-SCRN1, BioGFP-SCRN2, or BioGFP-SCRN3 and immunostained for SCRN1. Scale bar: 10 μm.

H Western blot of lysates from HEK293T cells expressing BioGFP-SCRN1, BioGFP-SCRN2, or BioGFP-SCRN2 and immunostained for indicated antibodies. Arrowheads represent (1) BioGFP-SCRN1 expression, (2) endogenous SCRN1 expression, (3) full-length BioGFP-SCRN proteins, and (4) N-terminal cleaved Bio GFP-SCRN2 and Bio GFP-SCRN3. Actin was used as loading control.

I Cortical neurons (DIV4) co-expressing RFP with pSuper empty vector (control) or SCRN1 shRNA #1. Scale bar: 10 μm.

J Quantifications of fluorescence intensity of endogenous SCRN1 in cortical neurons (DIV4) co-expressing RFP with pSuper empty vector (control) or SCRN1 shRNA #1, #2, or #3. $N = 2$, $n = 13$–14 cells.

Data information: Data represent mean ± SEM; $^{**}P < 0.01$; $^{***}P < 0.001$, by Mann–Whitney *U*-test.
Source data are available online for this figure.

 © 2019 The Authors

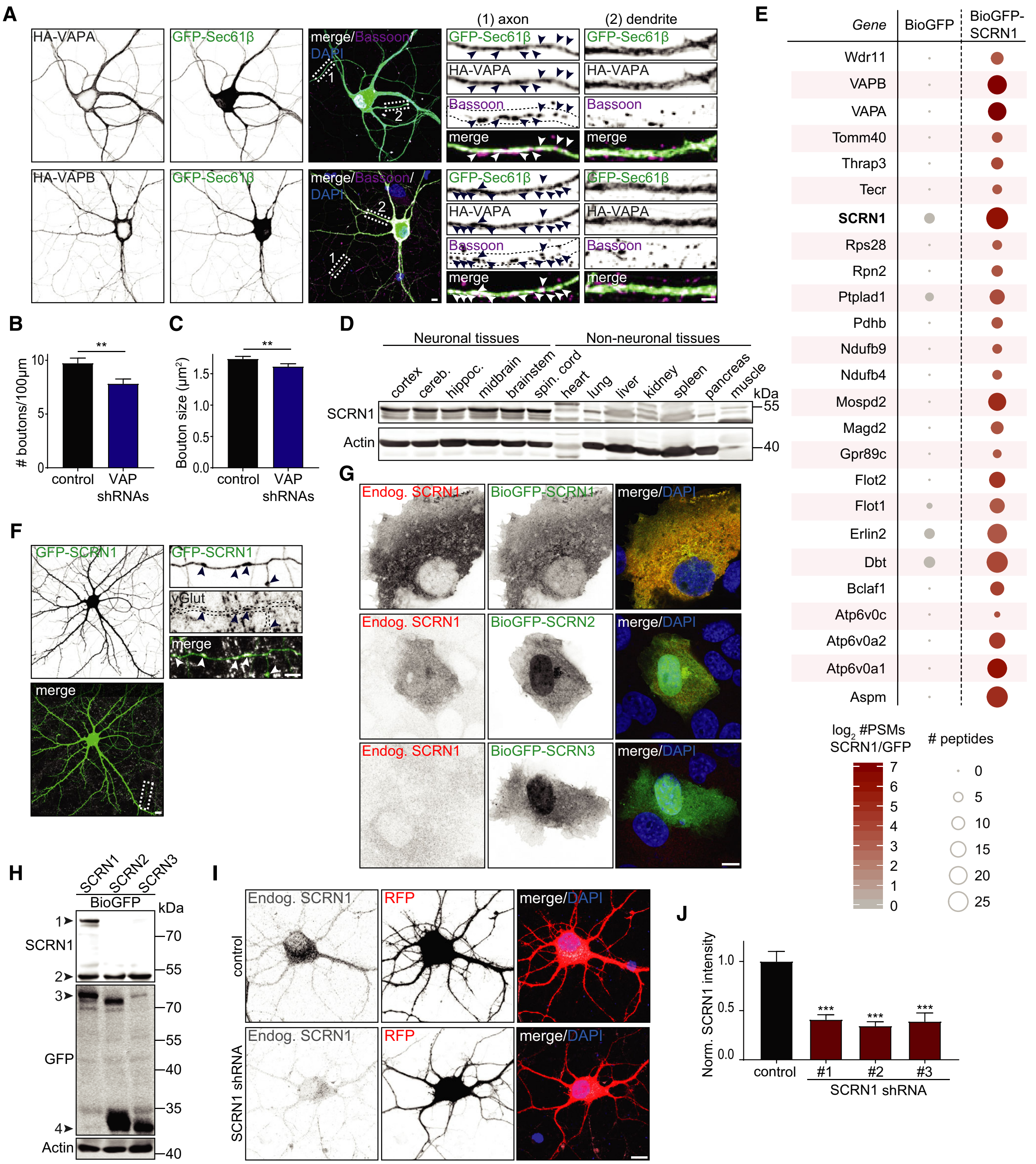

A
HA-VAPA
GFP-Sec61β
merge/Bassoon/DAPI
(1) axon
(2) dendrite
Bassoon
merge
HA-VAPB
B
boutons/100µm
control
VAP shRNAs
C
Bouton size (µm²)
D
Neuronal tissues
Non-neuronal tissues
cortex
cereb.
hippoc.
midbrain
brainstem
spin. cord
heart
lung
liver
kidney
spleen
pancreas
muscle
kDa
SCRN1
Actin
E
Gene
BioGFP
BioGFP-SCRN1
Wdr11
VAPB
VAPA
Tomm40
Thrap3
Tecr
SCRN1
Rps28
Rpn2
Ptplad1
Pdhb
Ndufb9
Ndufb4
Mospd2
Magd2
Gpr89c
Flot2
Flot1
Erlin2
Dbt
Bclaf1
Atp6v0c
Atp6v0a2
Atp6v0a1
Aspm
log2 #PSMs SCRN1/GFP
peptides
F
GFP-SCRN1
vGlut
merge
G
Endog. SCRN1
BioGFP-SCRN1
BioGFP-SCRN2
BioGFP-SCRN3
merge/DAPI
H
SCRN1
SCRN2
SCRN3
BioGFP
GFP
I
Endog. SCRN1
RFP
control
SCRN1 shRNA
J
Norm. SCRN1 intensity
#1
#2
#3

Figure EV1.

Figure EV2. SCRN1 does not exhibit proteolytic activity and its C-terminal is recruited to VAP MSP domain.

A Schematic illustration of autolytic protease activation of C69 family members.
B Sequence alignment of predicted proteolytic sites of SCRN family members according to the MEROPS database.
C Western blot of lysates from HEK293T cells expressing BioGFP-SCRN1-WT, BioGFP-SCRN1-C9A, BioGFP-SCRN2-WT, BioGFP-SCRN2-C12A, BioGFP-SCRN3-WT, or BioGFP-SCRN3-C6A. Arrowheads represent (1) full-length BioGFP-SCRN proteins and (2) N-terminal cleaved BioGFP-SCRN2 and BioGFP-SCRN3.
D Pull-down assay of HEK293T cells co-expressing Myc-VAPA with BioGFP, BioGFP-SCRN1-WT, BioGFP-SCRN1-C9A, BioGFP-SCRN1-N, or BioGFP-SCRN1-C.
E Pull-down assay of HEK293T cells co-expressing Myc-VAPB with BioGFP, BioGFP-SCRN1-WT, BioGFP-SCRN1-C9A, BioGFP-SCRN1-N, or BioGFP-SCRN1-C.
F Hippocampal neurons (DIV16) co-expressing HA-VAPB with GFP-SCRN1-N or GFP-SCRN1-C. Scale bars: 10 μm (full size) and 2 μm (zoom).
G COS7 cells co-expressing GFP-SCRN1-C with HA-VAPA or HA-VAPB. Scale bars: 10 μm (full size) and 5 μm (zoom).
H COS7 cells co-expressing HA-SCRN1 with GFP-VAPB-TM, GFP-VAPB-MSP-CC, or GFP-VAPB-MSP. Scale bars: 10 μm (full size) and 5 μm (zoom).
I COS7 cells co-expressing GFP-SCRN1-N with HA-VAPA or HA-VAPB. Scale bars: 10 μm (full size) and 5 μm (zoom).

Source data are available online for this figure.

 © 2019 The Authors

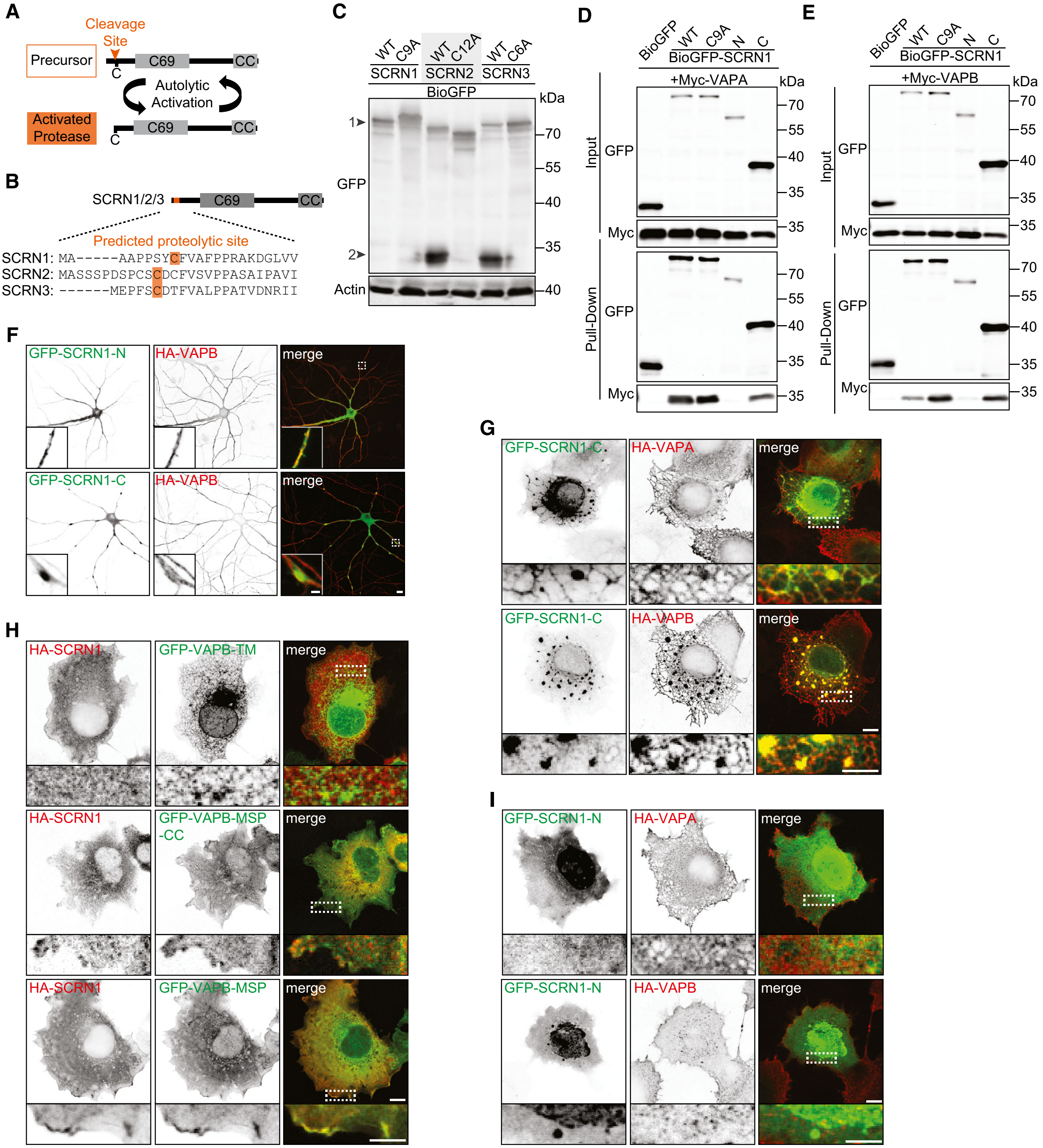
A
Cleavage Site
Precursor
C69
CC
Autolytic Activation
Activated Protease
B
SCRN1/2/3
Predicted proteolytic site
SCRN1: MA-----AAPPSYCFVAFPPRAKDGLVV
SCRN2: MASSSPDSPCSCDCFVSVPPASAIPAVI
SCRN3: ------MEPFSCDTFVALPPATVDNRII
C
WT C9A WT C12A WT C6A
SCRN1 SCRN2 SCRN3
BioGFP
kDa
GFP
Actin
D
BioGFP WT C9A N C
BioGFP-SCRN1
+Myc-VAPA
Input
Pull-Down
Myc
E
+Myc-VAPB
F
GFP-SCRN1-N
HA-VAPB
merge
GFP-SCRN1-C
G
HA-VAPA
H
HA-SCRN1
GFP-VAPB-TM
GFP-VAPB-MSP -CC
GFP-VAPB-MSP
I

Figure EV2.

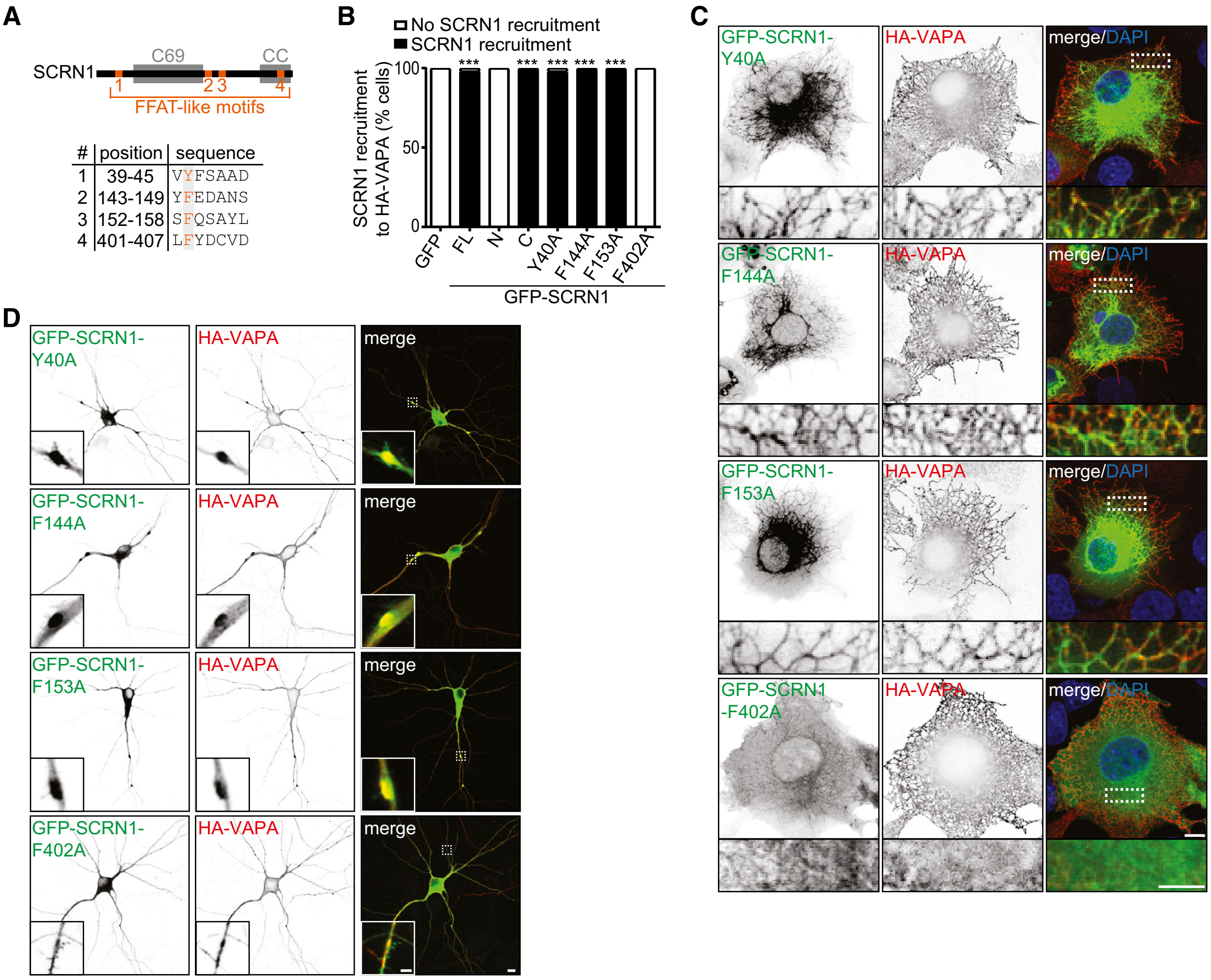

Figure EV3. VAP-SCRN1 interaction at the ER is mediated by a single FFAT-like motif.

A Schematic representation of the four potential FFAT-like motifs in SCRN1. Amino acid position number 2 (orange) of the FFAT-like motifs was mutated to alanine residues.

B Quantifications of SCRN1 recruitment to VAPA-positive structures in COS7 cells (%) co-expressing HA-VAPA with GFP or GFP-SCRN1, GFP-SCRN1-N, GFP-SCRN1-C, GFP-SCRN1-Y40A, GFP-SCRN1-F144A, GFP-SCRN1-F153A, or GFP-SCRN1-F402A. $N = 2–3$, $n = 39–59$.

C COS7 cells co-expressing HA-VAPA with GFP-SCRN1-Y40A, GFP-SCRN1-F144A, GFP-SCRN1-F153A, or GFP-SCRN1-F402A. Scale bars: 10 µm (full size) and 5 µm (zoom).

D Hippocampal neurons (DIV16) co-expressing HA-VAPA with GFP-SCRN1-Y40A, GFP-SCRN1-F144A, GFP-SCRN1-F153A, or GFP-SCRN1-F402A. Scale bars: 10 µm (full size) and 5 µm (zoom).

Data information: ***$P < 0.001$, by chi-square test with *post hoc* analysis including Bonferroni correction.

 © 2019 The Authors

Figure EV4. SCRN1 oligomerizes and regulates ER remodeling together with VAP.

A Quantifications of reticular localization of expressed VAPB in COS7 cells (%). Cells with non-reticular ER structures contained < 30% detectable ER tubules in cytoplasm. Gray bars represent non-reticular localization accompanied with impaired VAP–SCRN1 interaction. Left: co-expression GFP-SCRN1 with HA-VAPB or HA-VAPB-K87D/M89D (N = 2–3, n = 44–46). Right: co-expression of HA-VAPB with GFP or GFP-SCRN1, GFP-SCRN1-N, GFP-SCRN1-C, GFP-SCRN1-Y40A, GFP-SCRN1-F144A, GFP-SCRN1-F153A, or GFP-SCRN1-F402A (N = 2–3, n = 41–64).

B Quantifications of reticular localization in COS7 cells (%) co-expressing HA-VAPA with GFP or GFP-SCRN1, GFP-SCRN1-N, GFP-SCRN1-C, GFP-SCRN1-Y40A, GFP-SCRN1-F144A, GFP-SCRN1-F153A, or GFP-SCRN1-F402A. Cells with non-reticular ER structures contained < 30% detectable ER tubules in cytoplasm. Gray bars represent non-reticular localization accompanied with impaired VAP–SCRN1 interaction. N = 2–3, n = 39–62 cells.

C Quantifications of reticular localization of TagRFP-ER co-expressed with GFP, GFP-SCRN1, GFP-SCRN1-N, GFP-SCRN1-C, GFP-SCRN1-F402A, or GFP-SCRN1-C9A in COS7 cells. Gray bars represent non-reticular localization accompanied with impaired VAP–SCRN1 interaction. Cells with non-reticular ER structures contained < 30% detectable ER tubules in cytoplasm. N = 2–4, n = 58–120.

D Live COS7 cells co-expressing TagRFP-ER with GFP, GFP-SCRN1, or GFP-SCRN1-F402A. Scale bars: 10 μm (full size) and 2 μm (zoom).

E Pull-down assay of HEK293T cells co-expressing HA-SCRN1-N with BioGFP and BioGFP-SCRN1.

F Pull-down assay of HEK293T cells co-expressing HA-SCRN1-N with BioGFP, BioGFP-SCRN1-N, BioGFP-SCRN1-C, and BioGFP-SCRN1-F402A.

G Time-lapse of COS7 cells co-expressing TagRFP-ER and GFP, GFP-SCRN1, or GFP-SCRN1-F402A. Arrowheads mark ER tubule remodeling event, and arrows mark ER remodeling artifacts. Scale bar: 2 μm.

H ER nanostructures in somatic structures of hippocampal neurons (DIV18) co-expressing GFP-Sec61β with pSuper empty vector, SCRN1 shRNA, or VAPA/B shRNAs, and subjected to expansion microscopy. Left panels show maximum intensity projections of all Z-planes. Individual ER tubules and perinuclear sheets are shown in zooms of Z-plane #1 and Z-plane #2, respectively. Scale bars: 5 μm (full size) and 500 nm (zoom).

I ER nanostructures visualized with GFP-Sec61β in dendrites of hippocampal neurons (DIV18) immunostained for α-tubulin and co-expressed with pSuper empty vector, SCRN1 shRNA, or VAPA/B shRNAs, and subjected to expansion microscopy. Scale bars: 5 μm (full size) and 1 μm (zoom).

Data information: *P < 0.05; **P < 0.01; ***P < 0.001, by chi-square test (A–C) with *post hoc* analysis including Bonferroni correction (A, B).
Source data are available online for this figure.

© 2019 The Authors

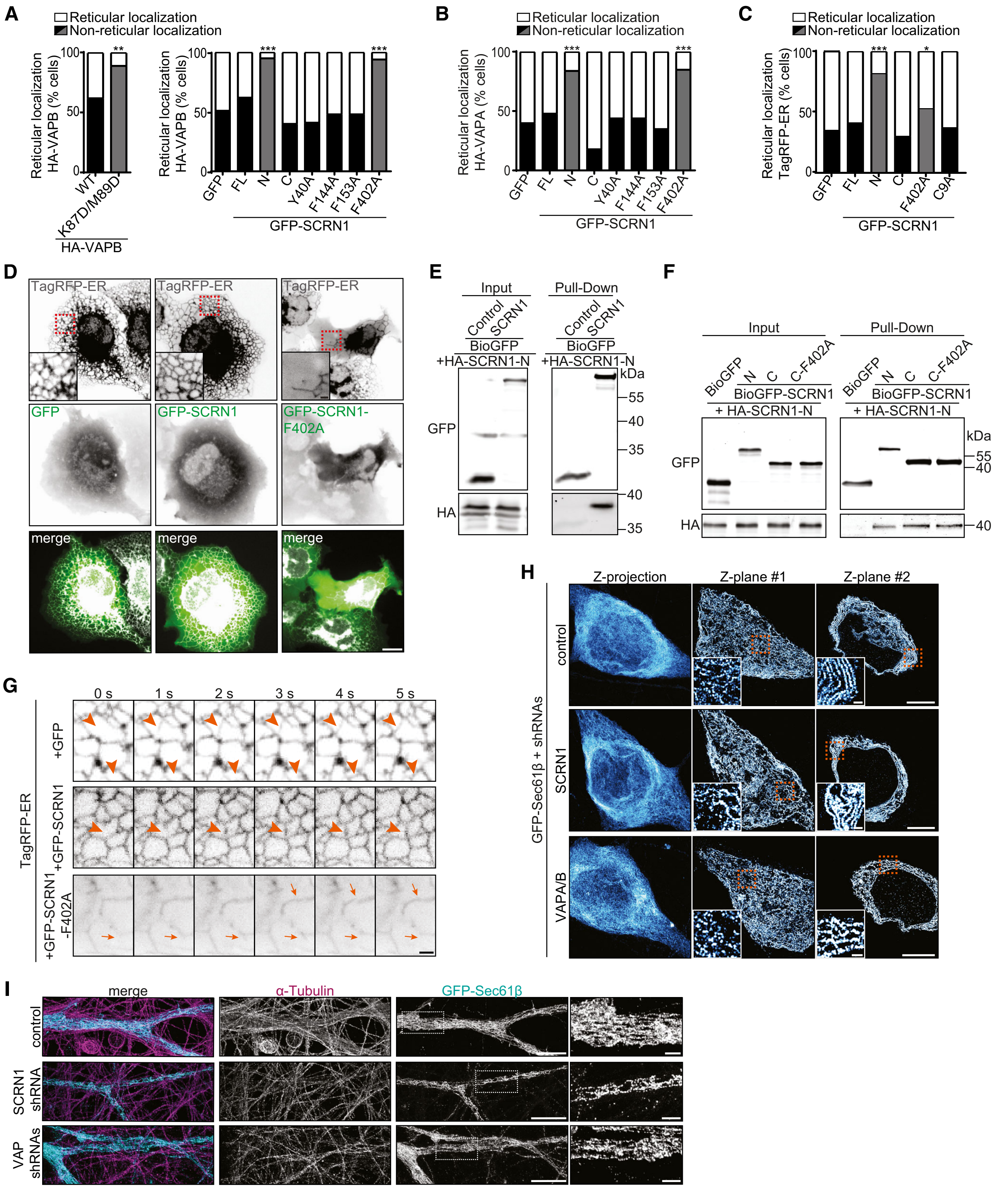

Figure EV4.

 © 2019 The Authors
