# Supplementary figures and images for: VAP‐SCRN1 interaction regulates dynamic endoplasmic reticulum remodeling and presynaptic function
Source: EMBO J. 2019 Aug 23;38(20):e101345. doi: 10.15252/embj.2018101345 (PMC6792018; doi:10.15252/embj.2018101345)

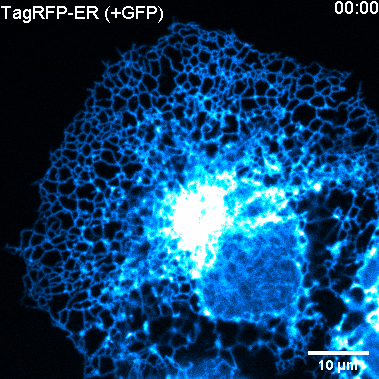

Supplement: Supplementary file 2 — Movie EV1 [file EMBJ-38-e101345-s002.zip › Movie_EV1/EMBOJ-2018-101345_Movie_EV1.gif]

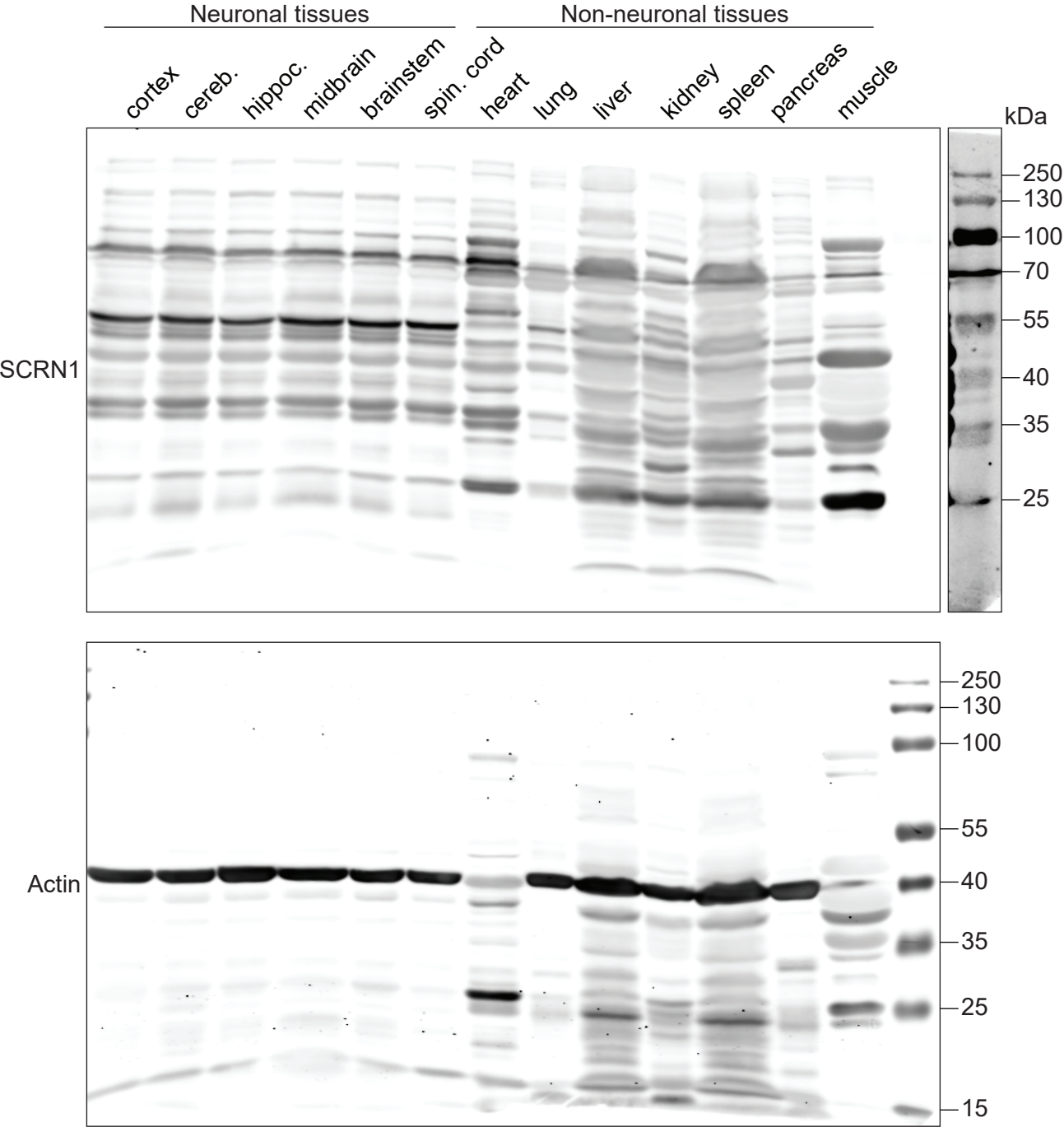

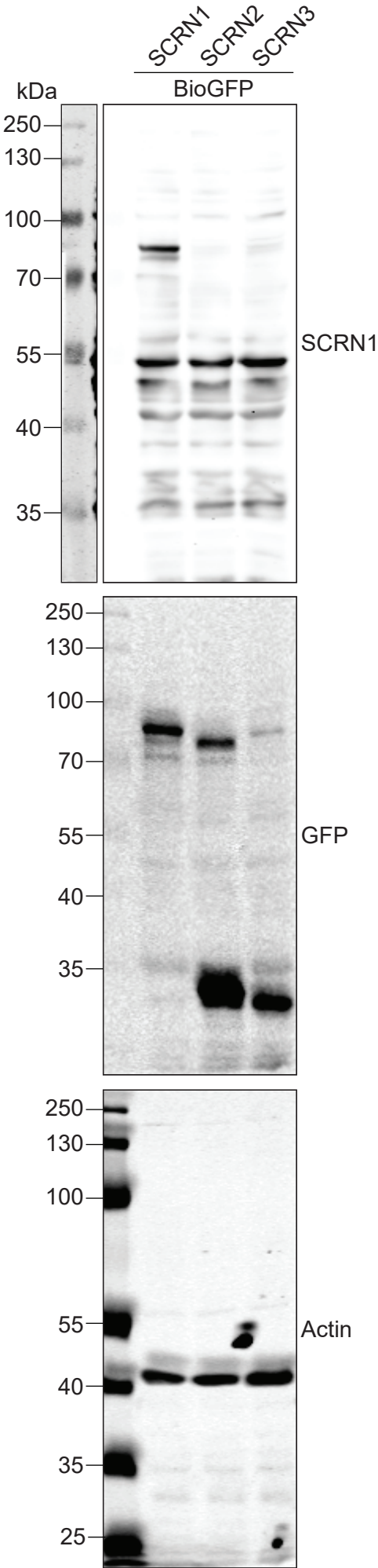

Supplement: Supplementary file 6 — Source Data for Expanded View [file EMBJ-38-e101345-s008.zip › Fig_EV_1_Source_Data.pdf]

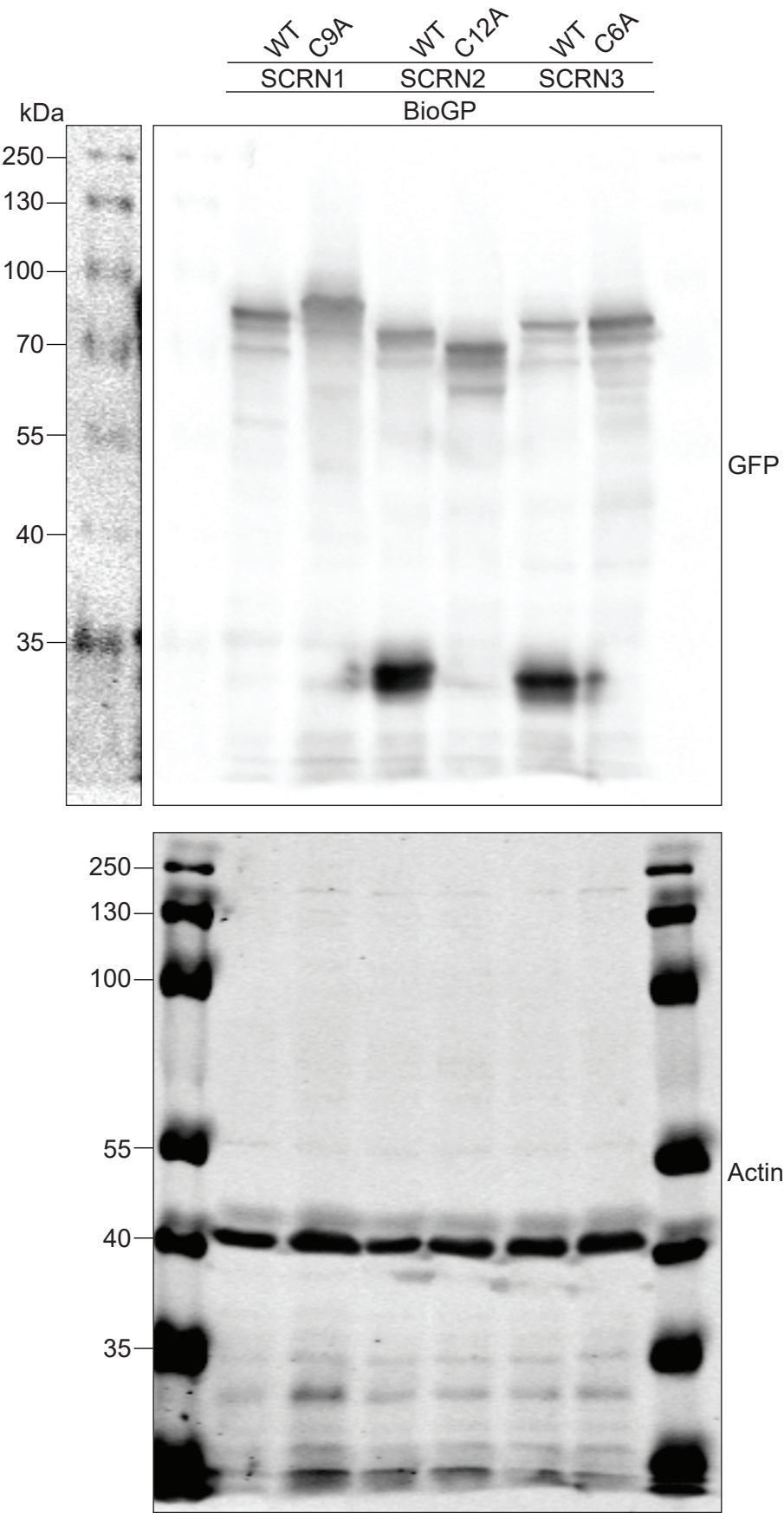

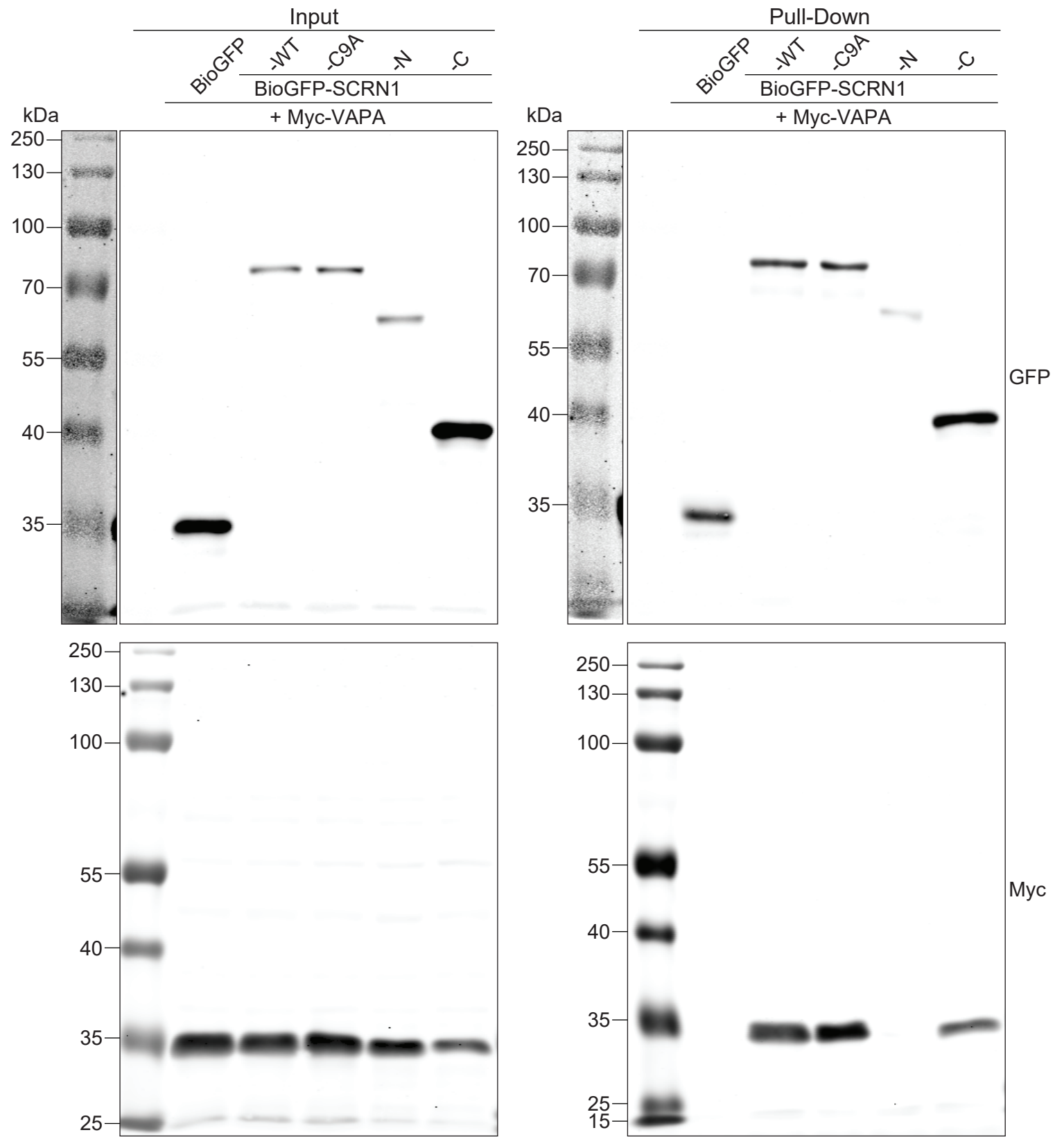

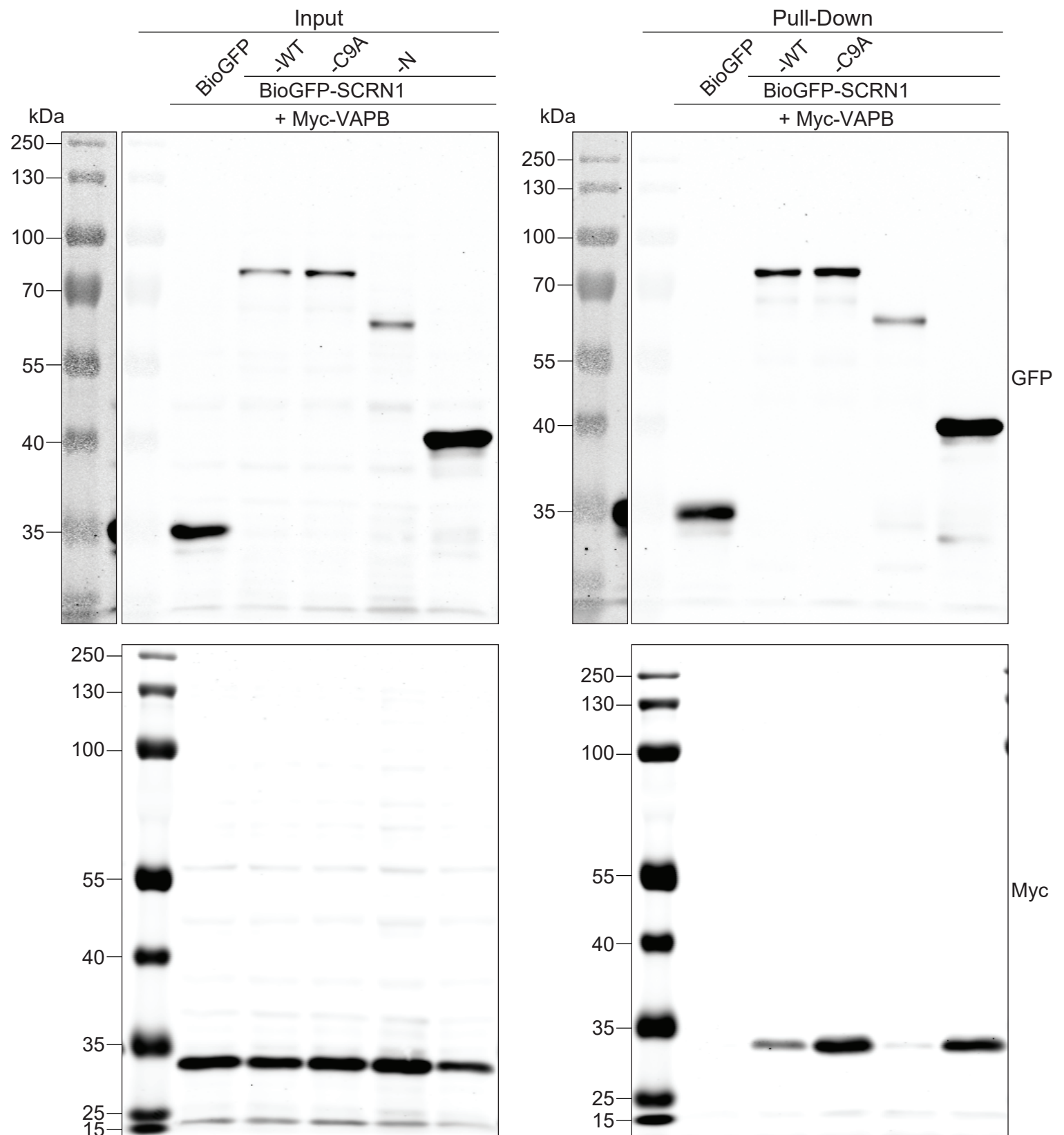

Supplement: Supplementary file 6 — Source Data for Expanded View [file EMBJ-38-e101345-s008.zip › Fig_EV_2_Source_Data.pdf]

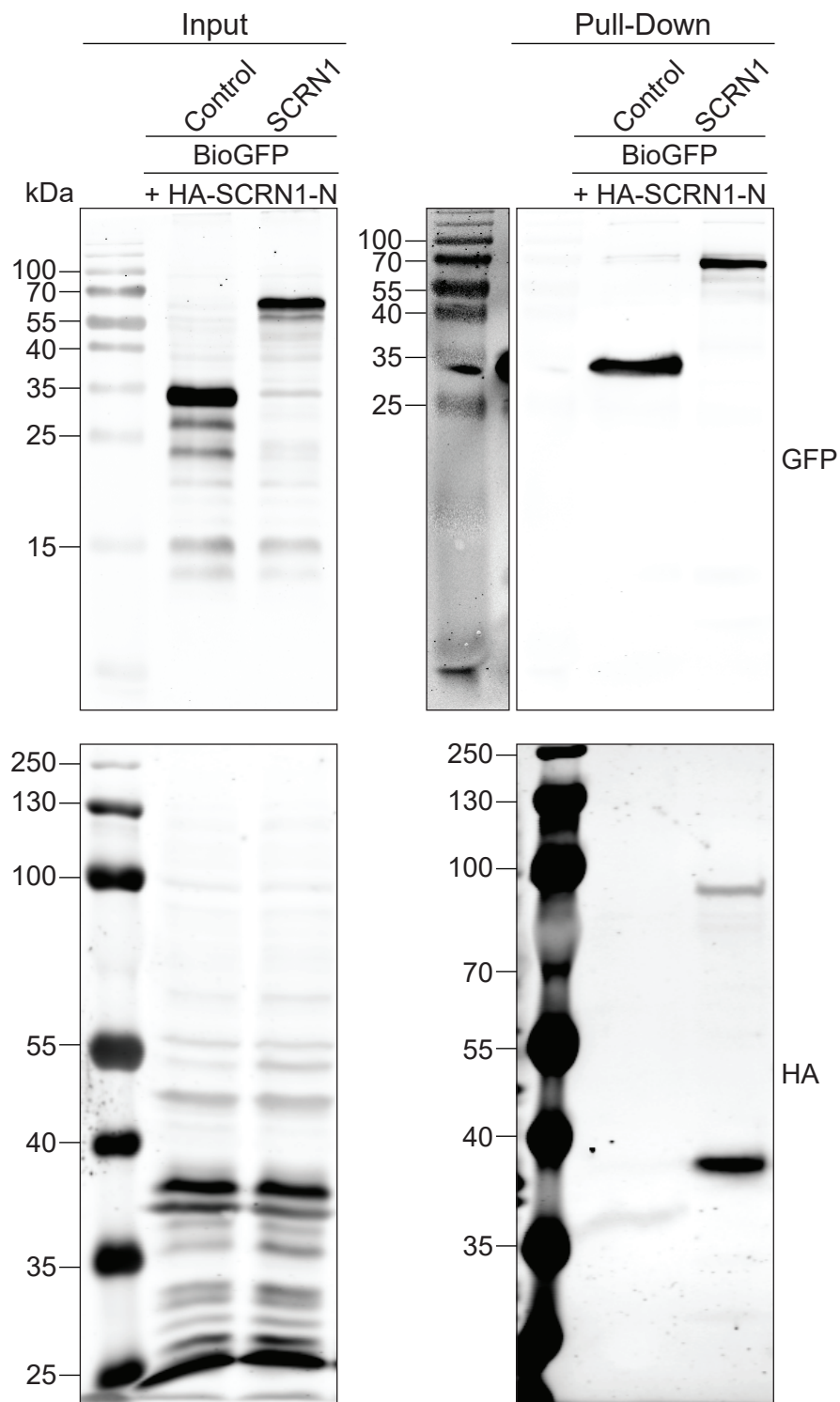

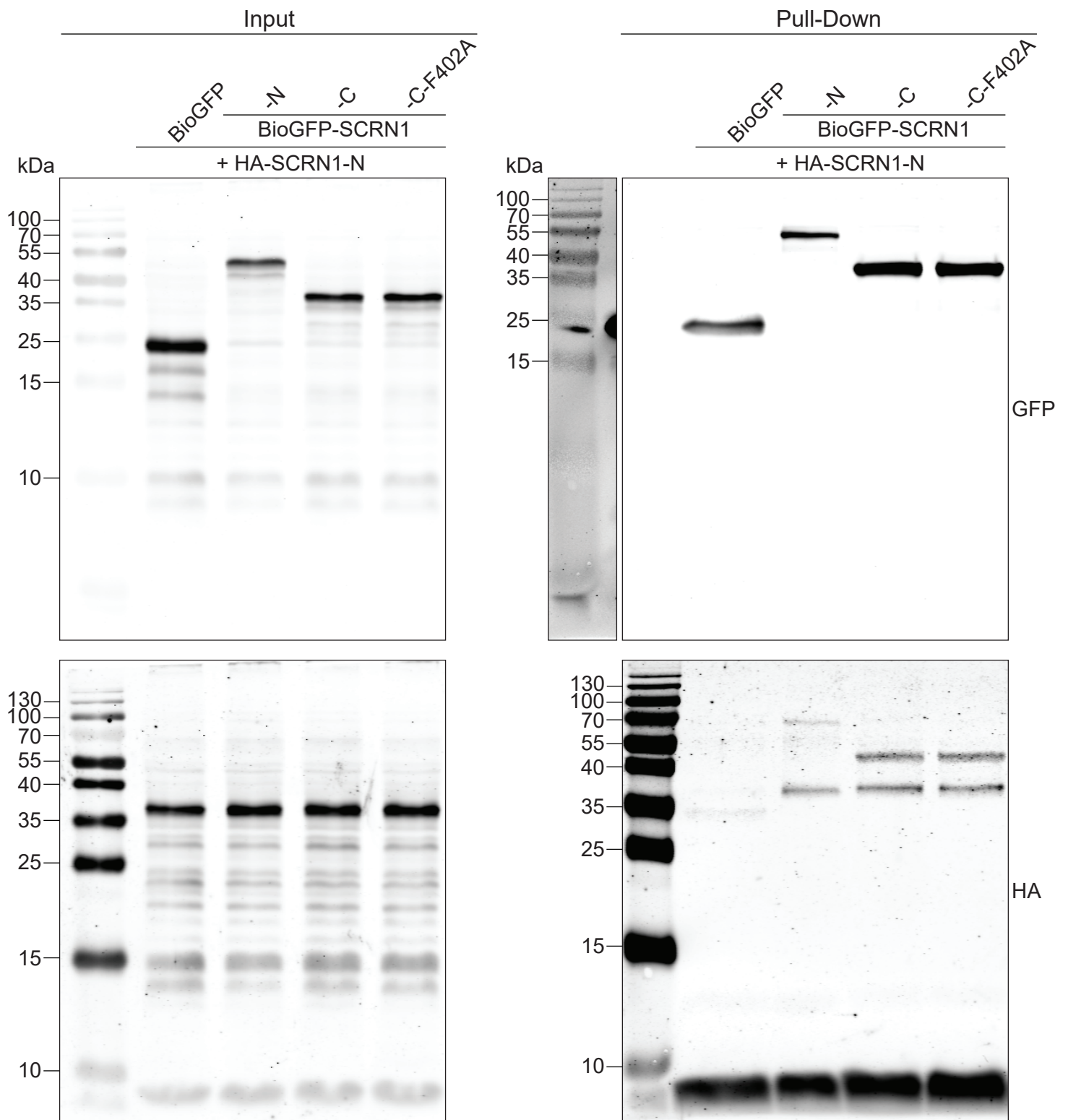

Supplement: Supplementary file 6 — Source Data for Expanded View [file EMBJ-38-e101345-s008.zip › Fig_EV_4_Source_Data.pdf]

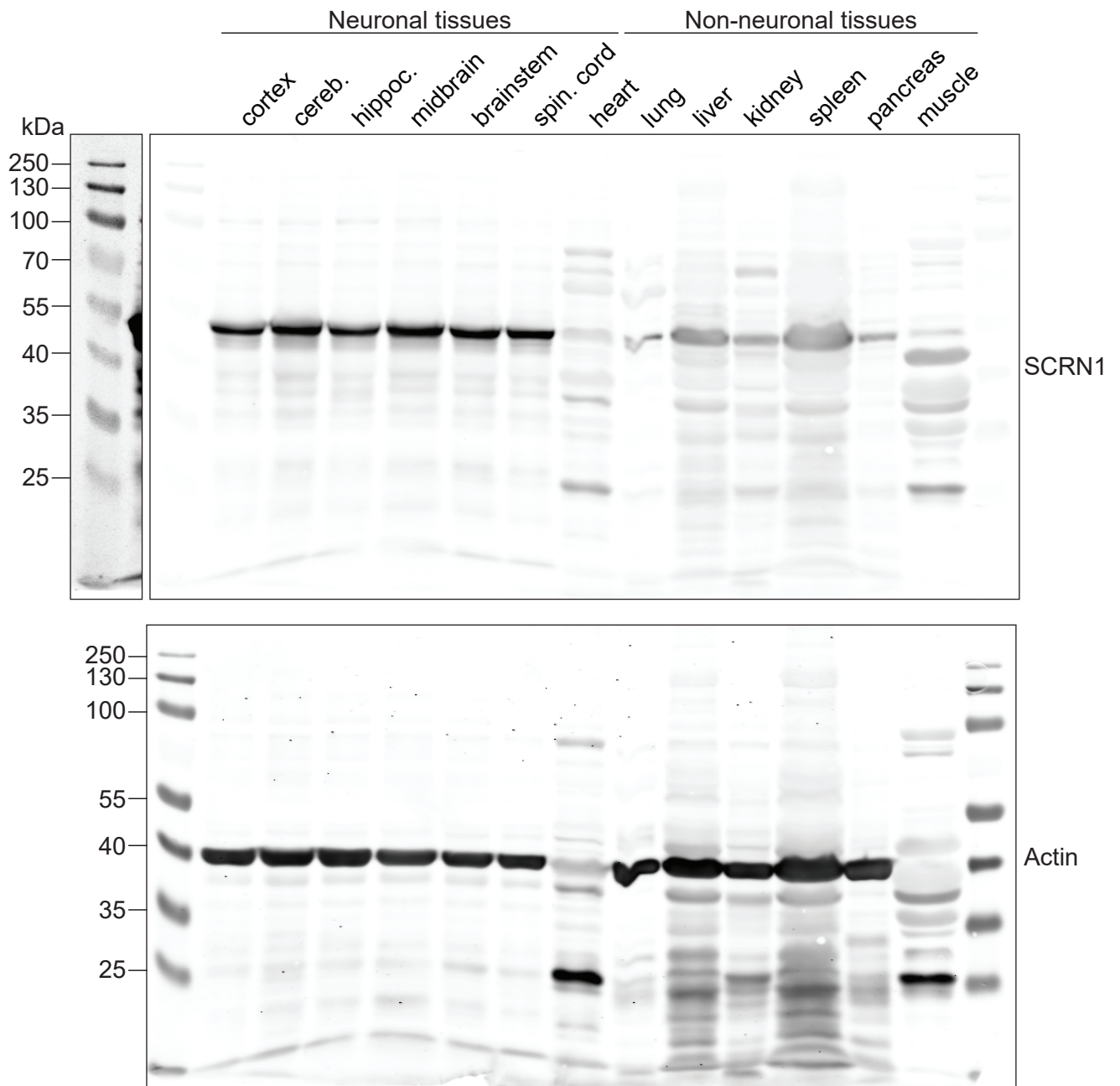

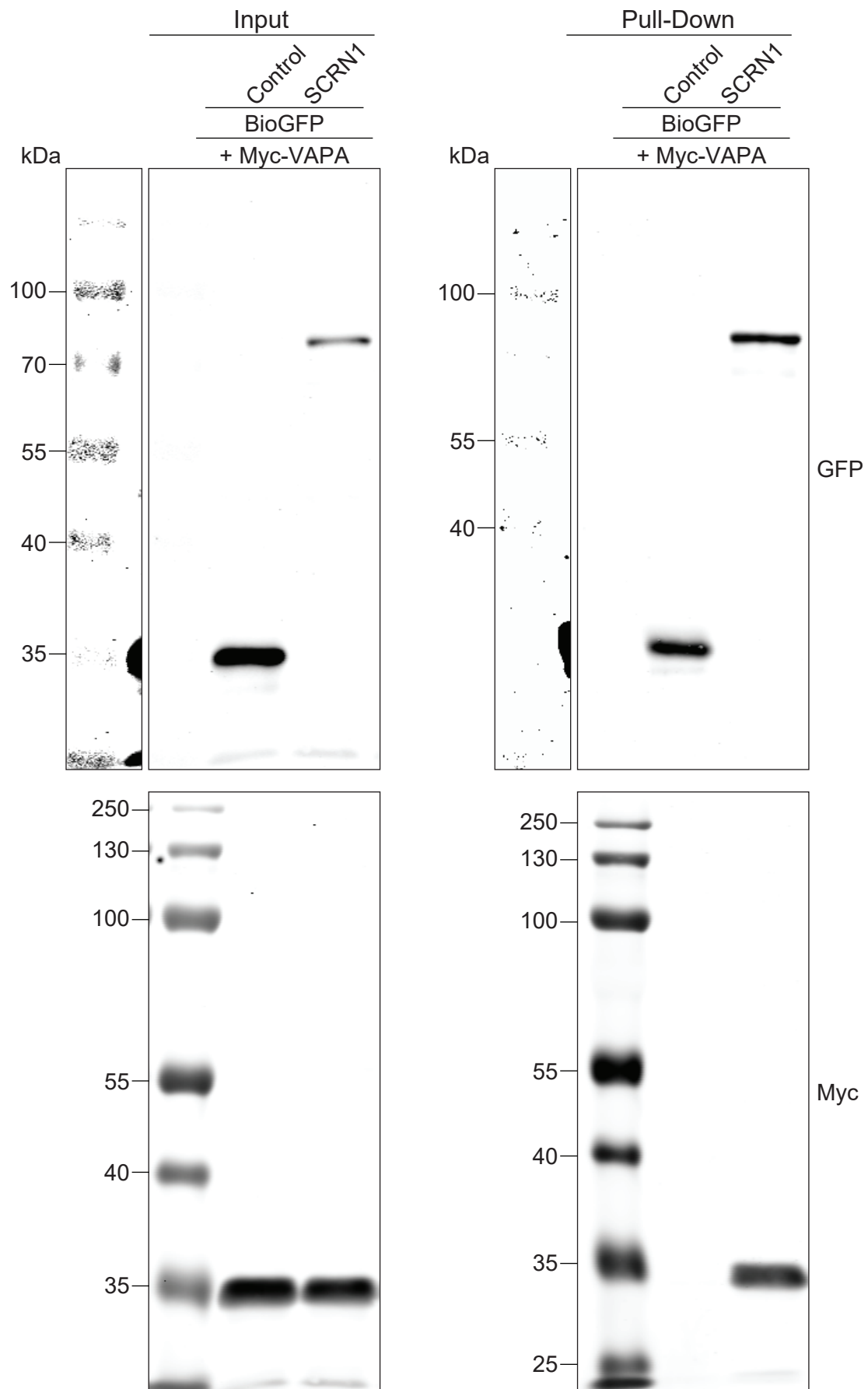

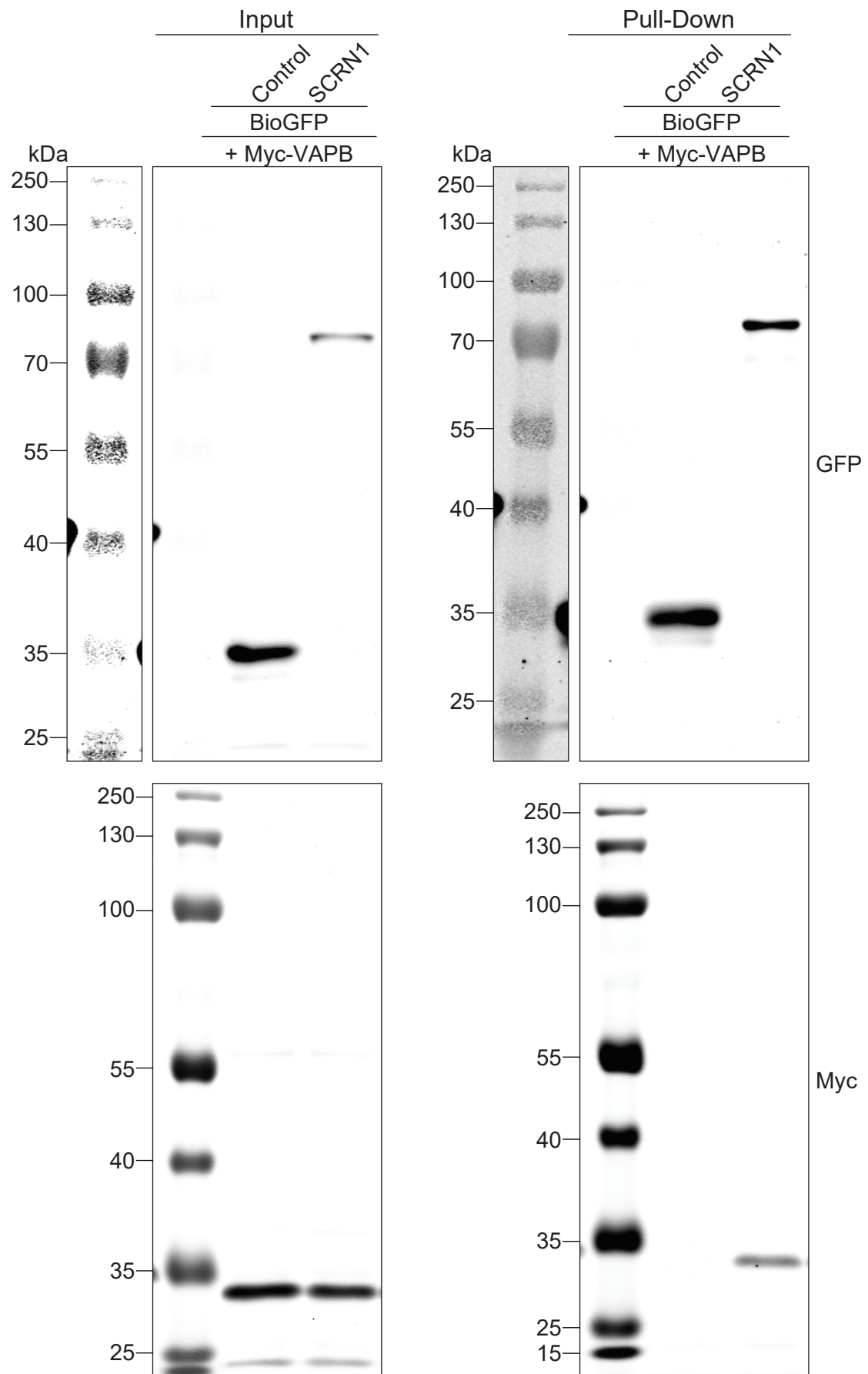

Supplement: Supplementary file 8 — Source Data for Figure 1 [file EMBJ-38-e101345-s006.pdf]

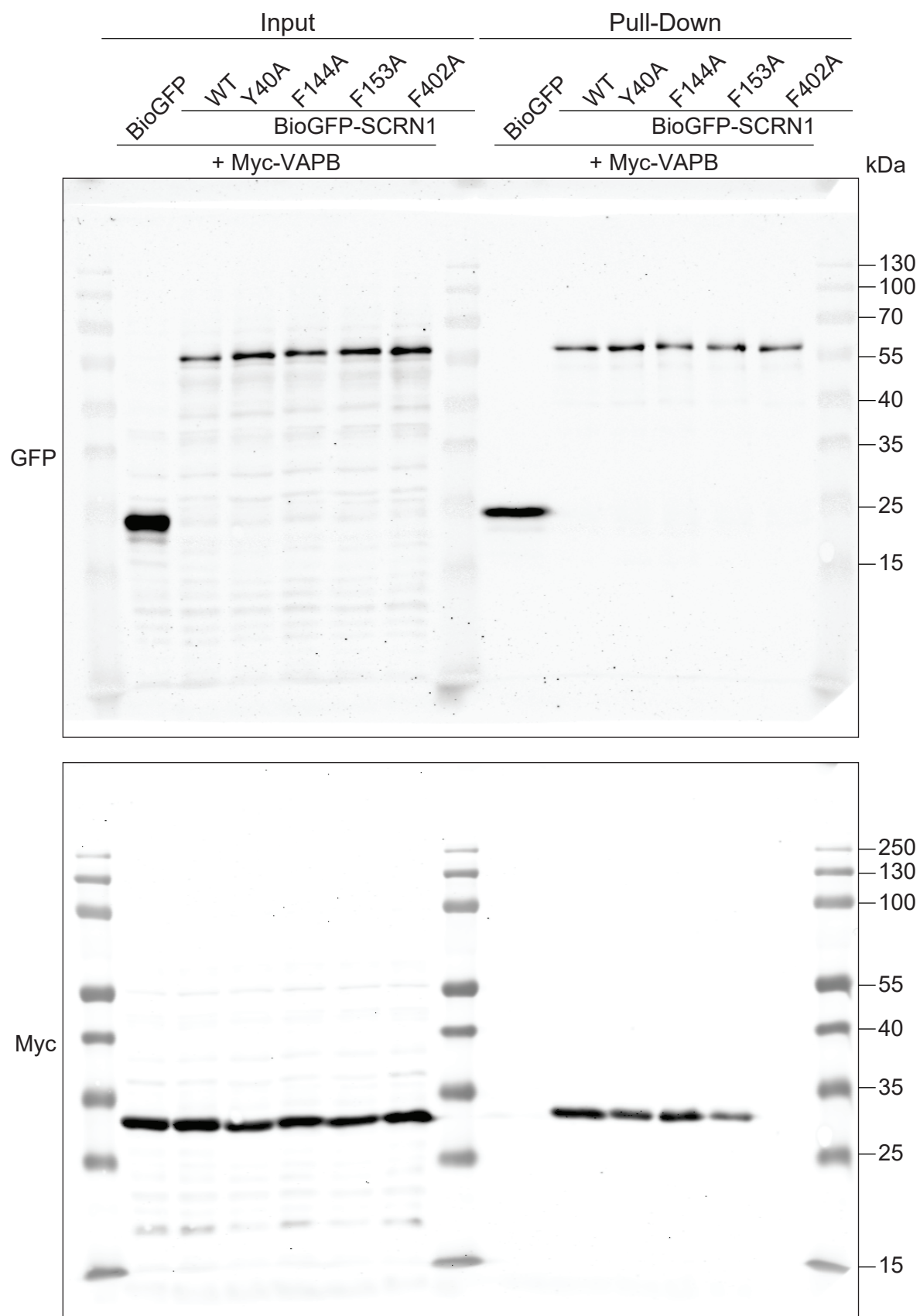

Supplement: Supplementary file 9 — Source Data for Figure 3 [file EMBJ-38-e101345-s007.pdf]
